# Impact of graft reperfusion on cardiac function assessed by transesophageal echocardiography during liver transplantation: an observational retrospective study

**DOI:** 10.1007/s10877-023-01110-5

**Published:** 2023-11-30

**Authors:** Susana González-Suárez, Matthew Corbett, Alberto Hernández-Martínez

**Affiliations:** 1 Department of Surgery, Universitat Autònoma de Barcelona, Unitat Docent Vall d´Hebron, Passeig Vall d´Hebron 119-129, Barcelona, 08035 Spain; 2 Department of Anesthesiology, Vall d´Hebron University Hospital, Passeig Vall d´Hebron 119-129, Barcelona, 08035 Spain; 3 Vall d´Hebron Institut de Recerca (VHIR), Cardiovascular diseases Research Group, Passeig Vall d´Hebron 119-129, Barcelona, 08035 Spain; 4 Department of Anesthesiology and Intensive Care, Grupo Policlínica, Ibiza. Islas Baleares. Vía Romana s/n, Ibiza, Baleares, 07800 Spain

**Keywords:** Transesophageal echocardiography, Liver transplantation, Hemodynamic instability, Ventricular dysfunction, Postreperfusion syndrome

## Abstract

Cardiovascular instability is common during the reperfusion phase of orthotopic liver transplantation (OLT), and some patients experience a postreperfusion syndrome (PRS). However, there are no reports comparing the cardiac dysfunction between patients with PRS and those without. Thus, the aim of this study was to evaluate cardiac dysfunction in patients exhibiting PRS. This observational retrospective study included 34 patients who underwent OLT and were monitored with transesophageal echocardiography (TEE). The right ventricular/left ventricular (RV/LV) end diastolic area, tricuspid annular plane systolic excursion (TAPSE), left ventricular ejection fraction (LVEF) by Simpson method, pulsed Doppler of the mitral valve, and tissue Doppler motion of the mitral annulus were determined. Echocardiographic measurements were registered at the beginning of surgery and at 1 and 30 min after vascular unclamping. Patients with PRS (PRS group) were identified, and their echocardiographic parameters of ventricular function were compared with those in patients without PRS (non-PRS group). To check the evolution of diastolic-systolic dysfunction, general linear model-repeated measures were estimated. No patient presented systolic/diastolic dysfunction on the basal echocardiogram. One minute after vascular unclamping, the incidence of RV dilation was 4.5 times greater in patients with PRS (*Cramer´s V* > 0.6), and the incidence of RV systolic dysfunction was 62.5% in patients with PRS compared to 15.40% in patients without PRS (*Cramer´s V* = 0.45). The incidence of LV systolic dysfunction was 25% in patients with PRS compared to 0% in those without (*Cramer´s V* = 0.45), and left ventricular diastolic dysfunction was 4.8 times greater in patients with PRS (*Cramer´s V* = 0.45). No patient presented diastolic dysfunction type III. There were significant differences between groups in the evolutionary pattern at 1 and 30 min after unclamping for RV dilation (*p* = 0.008) and for TAPSE (*p* = 0.014). Liver graft reperfusion may alter cardiac function. Cardiac dysfunction was more frequent in patients with PRS. These patients exhibited temporary dysfunction of the RV associated with a varying degree of LV diastolic-systolic dysfunction. *Trial registration*: clinicaltrials.gov (NCT05175534). January 03, 2022; “retrospectively registered”.

## Introduction

During orthotopic liver transplantation (OLT), several surgical stages can favor the appearance of hemodynamic instability [1–3]. One of these episodes is observed during the reperfusion phase; the surgical clamp is removed, leading to the release of cold, hyperkalemic fluid and acidic contents of the liver allograft into the circulation, causing a reduction in heart rate, an increase in pulmonary arterial pressure (PAP), a decrease in mean systemic blood pressure, and in some cases, temporary myocardial dysfunction. In this context, some patients may exhibit postreperfusion syndrome (PRS), defined as a decrease in the mean arterial pressure (MAP) ≥ 30% from baseline for more than 1 min and occurring within 5 min of unclamping of the inferior portal vein.

The visual assessment of the heart in this reperfusion stage is of extreme importance, and the characteristic echocardiographic features include acute ventricular systolic-diastolic dysfunction and new global or focal wall motion abnormalities [4–6].

There are no reports describing the use of transesophageal echocardiography (TEE) in association with PRS; thus, the aim of this study was to describe the grade of right-left cardiac dysfunction in patients exhibiting PRS.

## Methods

### Ethics statement and registration

This retrospective observational single-center study was approved by the Ethics Committee of Vall d’Hebron University Hospital, Barcelona, Spain (PR(AG)511/2019) and was registered at www.clinicaltrials.gov on January 03, 2022 (registration number: NCT05175534). All participants signed written informed consent for anesthesia, as well as echocardiography monitoring and recording of data, which was obtained for further analysis. All methods were carried out in accordance with relevant guidelines and regulations.

### Patient characteristics

Inclusion criteria included consecutive adult patients who underwent OLT from May 17, 2016 to November 14, 2019 (34 patients, 24% of the total OLT cases for this period in our hospital), who were monitored with TEE (they had no absolute contraindications such as the presence of esophageal stricture, cancer, diverticulum, recent esophageal surgery, or active upper gastrointestinal bleeding) and in whom the examination was performed by a TEE board-certified anesthesiologist. The exclusion criteria for the analysis were age under 18 years, hepatopulmonary syndrome, portopulmonary hypertension, previous cardiac surgeries, or cardiac diseases (heart failure, arrhythmias, left ventricular hypertrophy, diastolic-systolic ventricular dysfunction, moderate-severe valvular disease).

**Table 1:** Demographic characteristics, comorbidities, and their association with PRS

	Total patients n = 34	Non-PRS n = 26	PRS n = 8	*p*-value
Sex				
Woman	8 (23.5)	7 (26.9)	1 (12.5)	0.400
Man	26 (76.5)	19 (73.1)	7 (87.5)	
CHILD				
A	4 (11.8)	1 (3.8)	3 (37.5)	0.011
B	20 (58.8)	15 (57.7)	5 (62.5)	
C	10 (29.4)	10 (38.5)	0 (0)	
Cirrhosis				
Hepatitis B	3 (8.8)	3 (11.5)	0 (0)	0.717
Hepatitis C	10 (29.4)	6 (23.1)	4 (50)	
Alcoholic	12 (35.3)	9 (34.6)	3 (37.5)	
NASH	2 (5.9)	2 (7.7)	0 (0)	
Autoimmune	2 (5.9)	1 (3.8)	1 (12.5)	
Tumor	1 (2.9)	1 (3.8)	0 (0)	
PBC	1 (2.9)	1 (3.8)	0 (0)	
Cryptogenic	2 (5.9)	2 (7.7)	0 (0)	
Hyperoxaluria	1 (2.9)	1 (3.8)	0 (0)	
Renal disease	5 (14.7)	3 (11.5)	2 (25)	0.347
Diabetes	12 (35.3)	10 (38.5)	2 (25)	0.486
Hypertension	15 (44.1)	11 (42.3)	4 (50)	0.702
COPD	3 (8.8)	1 (3.8)	2 (25)	0.065
Cardiopathy	3 (8.8)	3 (11.5)	0 (0)	0.603
Hepatocarcinoma	16 (47.1)	10 (38.5)	6 (75)	0.070

Data are expressed as number (percentage)

NASH: nonalcoholic steatohepatitis, PBC: primary biliary cirrhosis, COPD: chronic obstructive pulmonary disease

### Anesthesia

After establishing non-invasive monitoring, anesthesia induction was performed with the administration of 2 µg/kg of fentanyl, 2 mg/kg of propofol, and 0.5 mg/kg of atracurium. After tracheal intubation, mechanical ventilation was started (55–60% oxygen–air mixture) and adjusted to maintain an end-tidal carbon dioxide concentration between 33 and 38 mmHg. Anesthesia was maintained with a desflurane (3-6%) and fentanyl (2 µg/kg^/^h) infusion and atracurium infusion (0.4 mg/kg/h). Hemodynamic parameters were obtained by Swan Ganz catheter placement or by the FloTrac/Vigileo™ system (Edwards Lifesciences, Irvine, CA). A TEE probe was inserted from the beginning of surgery until the first thirty minutes of the neohepatic phase (in cases of hemodynamic instability, it was removed at the end of surgery). Electrolytes and arterial blood gases were monitored and corrected throughout the surgery. Anesthetic management during the anhepatic phase focused on the maintenance of cardiac preload and correction of arterial blood gas and electrolyte imbalances. A base deficit greater than 10 mmol/L was treated with sodium bicarbonate. An ionized calcium level of < 4 mg/dL was treated with calcium chloride, and hyperkalemia (> 5 mmol/L) was treated with insulin and glucose. Noradrenaline infusion was administered when the systolic arterial blood pressure remained below 90 mmHg.

**Table 2:** Frequency of ventricular dysfunction measured at 1 and 30 min after graft reperfusion

	Total n = 34	Non-PRS n = 26	PRS n = 8	*P-*value Chi2	*V* de Cramer
RV/LV end diastolic area-1 min					
< 0.6	22 (64.7)	21 (80.8)	1 (12.5)	0.000	0.606
≥ 0.6	12 (35.3)	5 (19.2)	7 (87.5)		
TAPSE-1 min					
< 17 mm	9 (26.5)	4 (15.4)	5 (62.5)	0.008	0.453
≥ 17 mm	25 (73.5)	22 (84.6)	3 (37.5)		
LVEF-1 min					
< 53%	2 (5.9)	0 (0)	2 (25)	0.009	0.451
≥ 53%	32 (94.1)	26 (100)	6 (75)		
LV diastolic dysfunction-1 min	5 (14.7)	2 (7.7)	3 (37.5)	0.029	0.457
RV/LV end diastolic area-30 min					
< 0.6	33 (97.1)	26 (100)	7 (87.5)	0.067	0.314
≥ 0.6	1 (2.9)	0 (0)	1 (12.5)		
TAPSE-30 min					
< 17 mm	3 (8.8)	3 (11.5)	0 (0)	0.314	0.173
≥ 17 mm	31 (91.2)	23 (88.5)	8 (100)		
LVEF-30 min					
< 53%	0 (0)	0 (0)	0 (0)		
≥ 53%	34 (100)	26 (100)	8 (100)	-	.
**LV diastolic dysfunction-30 min**	1 (2.9)	0 (0.0)	1 (12.5)	0.067	0.314

Data are expressed as number (percentage). The magnitude of the effect for Cramer’s V was: 0.00–0.09 as negligible, 0.10–0.29 as low, 0.30–0.49 as medium and from 0.50 as high

PRS: postreperfusion syndrome, RV: right ventricle, LV: left ventricle, TAPSE: tricuspid annular plane systolic excursion, LVEF: left ventricle ejection fraction

### Surgical technique

Liver allografts were preserved in a cold University of Wisconsin solution. Anastomosis of the liver graft was performed using the piggyback technique with or without temporary portocaval shunting. Before completing the hepatic vein anastomosis, the liver graft was perfused with albumin through the portal vein. All patients were transported to intensive care unit (ICU), postoperatively.

**Table 3:** Hemodynamic parameters in patients with postreperfusion syndrome (PRS) and without postreperfusion syndrome

		Basal	1 min	30 min	
		Mean (CI 95%)	Mean (CI 95%)	Mean (CI 95%)	*p-value*
CVP	Non-PRS	5,80 (4.09,7.51)	6,96 (5.31,8.61)	7,23 (5.56,8.89)	0.574
	PRS	7,50 (4.41,10.58)	7,50 (4.41,10.58)	8,87 (5.87,11.87)	
PCP	Non-PRS	11.74 (9.31,14.16)	13.47 (11.14,15.81)	14.65 (12.47,16.82)	0.635
	PRS	14.33 (9.58,19.08)	15.50 (10.92,20.07)	14.00 (9.74,18.25)	
SVRI	Non-PRS	1620.61 (1461.17,1780.05)	1474.92 (1324.40,1625.44)	1625.57 (1470.71,1780.43)	0.56
	PRS	1587.88 (1300.44,1875.31)	1297.37 (1026.01,1568.73)	1680.03 (1400.85,1959.21)	
MAP	Non-PRS	76.54 (72.09,80.99)	63.08 (59.90,66.25)	81.46 (78.44,84.49)	0.000
	PRS	74.38 (66.35,82.40)	40.50 (34.77,46.23)	76.25 (70.80,81.70)	
CI	Non-PRS	3.72 (3.26,4.18)	3.24 (2.87,3.61)	3.84 (3.44,4.25)	0.021
	PRS	3.51 (2.69,4.34)	2.10 (1.44,2.76)	3.42 (2.69,4.14)	
HR	Non-PRS	75.50 (69.36,81.64)	73.85 (68.08,79.61)	76.46 (71.83,81.09)	0.413
	PRS	82.25 (71.18,93.32)	76.50 (66.10,86.90)	82.25 (73.91,90.59)	

CVP: central venous pressure, PCP: pulmonary capillary pressure, SVRI: systemic vascular resistance index, MAP: mean arterial pressure, CI: cardiac index, HR: heart rate

### Echocardiographic methods

A TEE scan was performed for intraoperative monitoring at the beginning of surgery, at 1 min and at 30 min, in all patients after vascular unclamping and in cases of hemodynamic instability (MAP minor than 60 mmHg).

The systolic evaluation for the LV was performed by the Simpson method using midesophageal 4-chamber (ME 4 C) view; a value < 53% was considered pathological [7].

The LV diastolic function was assessed in ME 4 C view by pulsed Doppler of the mitral valve at the level of the leaflet tips (E/A), and by tissue doppler of the mitral annulus at the septal (e´ septal) and lateral (e´ lateral) levels [8]. Biplane left atrial (LA) volume was estimated using Simpson´s method [9]. RV systolic function was measured with TAPSE; a value of less than 17 mm was considered pathological [7]. The presence of RV dilation was defined as an RV/LV end diastolic area ≥ 0.6 at ME 4 C [10–13].

**Table 4:** Evolution and comparation of systolic and diastolic function parameters between groups

		Basal Mean (CI95%)	1 min Mean (CI95%)	30 min Mean (CI95%)	1 min-Basal Mean (CI95%)	Variation%	30 min-Basal Mean (CI95%)	Variation%	*P*-Value
RV/LV	Total	0.493 (0.458,0.528)	0.663 (0.616,0.710)	0.513 (0.487,0.539)	0.170 (0.112,0.227)	34.5%**	0.020 (-0.023,0.063)	4.1%	0.000**
	Non-PRS	0.524 (0.490,0.558)	0.602 (0.557,0.648)	0.500 (0.475,0.526)	0.078 (0.022,0.134)	14.9%**	-0.024 (-0.065,0.018)	-4.6%	0.008**
	PRS	0.463 (0.402,0.523)	0.724 (0.641,0.806)	0.526 (0.481,0.572)	0.261 (0.161,0.362)	56.4%**	0.064 (-0.011,0.139)	13.8%*	
TAPSE (mm)	Total	19.120 (18.399,19.841)	16.764 (16.161,17.368)	18.731 (18.021,19.440)	-2.356 (-3.278, -1.433)	-12.3%**	-0.389 (-1.381,0.602)	-2.0%	0.000**
	Non-PRS	18.615 (17.916,19.315)	17.654 (17.068,18.239)	18.962 (18.273,19.650)	-0.962 (-1.857, -0.067)	-5.2%*	0.346 (-0.616,1.308)	1.9%	0.014*
	PRS	19.625 (18.365,20.885)	15.875 (14.819,16.931)	18.500 (17.259,19.741)	-3.750 (-5.363, -2.137)	-19.1%**	-1.125 (-2.860,0.610)	-5.7%	
LVEF (%)	Total	64.053 (61.785,66.321)	60.111 (57.889,62.332)	61.620 (59.662,63.578)	-3.942 (-7.056, -0.829)	-6.2%*	-2.433 (-5.373,0.507)	-3.8%	0.045*
	Non-PRS	63.731 (61.530,65.931)	61.846 (59.691,64.001)	62.115 (60.216,64.015)	-1.885 (-4.905,1.136)	-2.9%	-1.615 (-4.468,1.237)	-2.5%	0.416
	PRS	64.375 (60.408,68.432)	58.375 (54.490,62.260)	61.125 (57.701,64.549)	-6.000 (-11.446, -0.554)	-9.3%*	-3.250 (-8.392,1.892)	-5.0%	
E/A	Total	1.252 (1.201,1.303)	1.475 (1.384,1.565)	1.305 (1.238,1.372)	0.223 (0.120,0.325)	17.8%**	0.053 (-0.029,0.136)	4.2%	0.000**
	Non-PRS	1.261 (1.212,1.310)	1.412 (1.324,1.499)	1.261 (1.196,1.326)	0.150 (0.051,0.250)	11.9%**	0.000 (-0.080,0.080)	0.0%	0.281
	PRS	1.243 (1.154,1.331)	1.538 (1.379,1.696)	1.349 (1.232,1.466)	0.295 (0.116,0.474)	23.7%**	0.106 (-0.038,0.251)	8.5%	
E/e´	Total	7.146 (6.911,7.382)	10.251 (9.014,11.488)	8.130 (7.451,8.810)	3.105 (1.849, 4.360)	43.5%**	0.984 (0.270,1.698)	13.8%**	0.000**
	Non-PRS	6.943 (6.714,7.171)	7.079 (5.879,8.279)	7.103 (6.444,7.763)	0.137 (-1.082,1355)	1.9%	0.161 (-0.532,0.853)	2.3%	0.000**
	PRS	7.350 (6.939,7.761)	13.423 (11.259,15.586)	9.157 (7.969,10.346)	6.072 (3876,8.269)	82.6%**	1.807 (0.559,3.056)	24.6%**	
TR (m/s)	Total	0.224 (0.091,0.356)	1.160 (0.821,1.498)	0.333 (0.108,0.557)	0.936 (0.576,1.296)	417.9%**	0.109 (-0.148,0.366)	48.7%	0.000**
	Non-PRS	0.135 (0.006,0.263)	0.719 (0.391,1.047)	0.115 (-0.102,0.333)	0.585 (0.235,0.934)	433.3%**	-0.019 (-0.268,0.230)	-14.1%	0.009**
	PRS	0.313 (0.080,0.545)	1.600 (1.009,2.191)	0.550 (0.158,0.942)	1.288 (0.658,1.917)	411.5%**	0.238 (-0.212,0.687)	76%	
LA vol. índex (mL/m^2^)	Total	25.558 (24.246,26.898)	27.058 (25.271,28.845)	26.053 (24.631,27.475)	1500 (-0.678, 3.678)	5.9%	0.495 (-1.402,2.393)	1.9%	0.392
	Non-PRS	25.115 (23.843,26.388)	26.115 (24.382,27.849)	25.231 (23.851,26.610)	1.000 (-1.113,3.113)	3.9%	0.115 (-1.725,1.956)	0.5%	0.398
	PRS	26.000 (23.706,28.294)	28.000 (24.875,31.125)	26.875 (24.388,29.362)	2.000 (-1.809,5.809)	7.7%	0.875 (-2.443,4.193)	3.4%	

General linear models (GLM) repeated measures to determine significant variations in the parameters of ventricular dysfunction over time and the influence of PRS. On the left of the table (non-shaded table), data are shown as mean (estimated marginal according to GLM) and 95% confidence interval (CI) at basal, 1 min, and 30 min after graft reperfusion. On the right of the table (shaded table), data are expressed as means of units of variation (95% CI) and percentages of variation in relation to baseline values at 1 min and at 30 min after vascular unclamping

RV: right ventricle, TAPSE: tricuspid annular plane systolic excursion, LVEF: left ventricular ejection fraction, TR: tricuspid regurgitation, LA: left atrial

*P-value <0.05 (significant result at 5%), **p-value <0.01 (significant result at 1%)

ME 4 C was performed to detect possible venous air embolism and thrombus and to evaluate the interaction of the left and right ventricles [14].

The various complications of TEE were also registered after orotracheal extubation, such as dental trauma, variceal bleeding, esophageal trauma, and recurrent laryngeal nerve injury.

### Study outcome

Our study outcome was to describe the right and left cardiac dysfunction at the reperfusion phase between patients with and without PRS.

### Data collection

Age, sex, Child-Pugh classification, cirrhosis etiology, presence of hepatocarcinoma, hypertension, diabetes mellitus, renal disease, chronic obstructive pulmonary disease, and cardiopathy were recorded at baseline. The following data were analyzed at the beginning of surgery, one min after reperfusion, and 30 min after reperfusion: MAP, heart rate (HR), cardiac index (CI), central venous pressure (CVP), pulmonary capillary pressure (PCP), SVRI (systemic vascular resistance index), LA volume index, RV end diastolic area, LV end diastolic area, TAPSE, tricuspid regurgitation (TR) velocity, LVEF, E/a (pulsed Doppler of the mitral valve), and e´ (tissue Doppler of the mitral annulus). The data on need for vasoactive drugs during surgery, as well as postoperative data on graft rejection, the need for second surgery after liver transplant (bleeding), the need for re-transplantation, renal dysfunction (glomerular filtration < 60 mL/min/1.73 m^2^), days of hospitalization, mortality, and complications associated with TEE were collected from the patients’ medical records during hospital admission.

### Statistical analysis

For categorical variables, frequencies and percentages are shown for the total sample and the two groups (case/control). The differences between groups were evaluated with Pearson’s nonparametric Chi-squared test; Fisher’s exact test was applied only in the case of 2 × 2 tables. For continuous variables, descriptive values of mean and standard deviation were displayed. The differences between groups were evaluated using the Mann-Whitney test (nonparametric) or Student´s test for two independent samples (parametric) based on the normality of the variables evaluated using the Shapiro-Wilks test. To check the evolution of diastolic/systolic dysfunction, general linear model (GLM)-repeated measures were estimated with robust covariances. All contrasts were accompanied by the effect size estimator to complete the interpretation of the results (*Cramer’s V* for categorical variables). The criteria for classifying the magnitude of the effect for *Cramer’s V* were as follows: negligible, 0.00-0.09; low, 0.10–0.29; medium, 0.30–0.49; and high, 0.50. The significance level used in the analyzes was set at 5% (*α =* 0.05).

## Results

Thirty-four patients were enrolled in the study, including 26 men and eight women. The mean age of patients with PRS (n = 8) was 58.42 ± 10.24 years, and that of patients without PRS (n = 26) was 61 ± 8.32 years, *p* = 0.460.

The baseline characteristics of the patients are shown in Table 1. Differences between groups were observed for Child-Pugh score.

No patient presented systolic/diastolic dysfunction on the preoperative echocardiogram. In six patients, cardiac monitoring with TEE was not possible in the transgastric short axis view (TG mid SAX) due to retraction of the stomach by surgical separators.

**Fig. 1:**
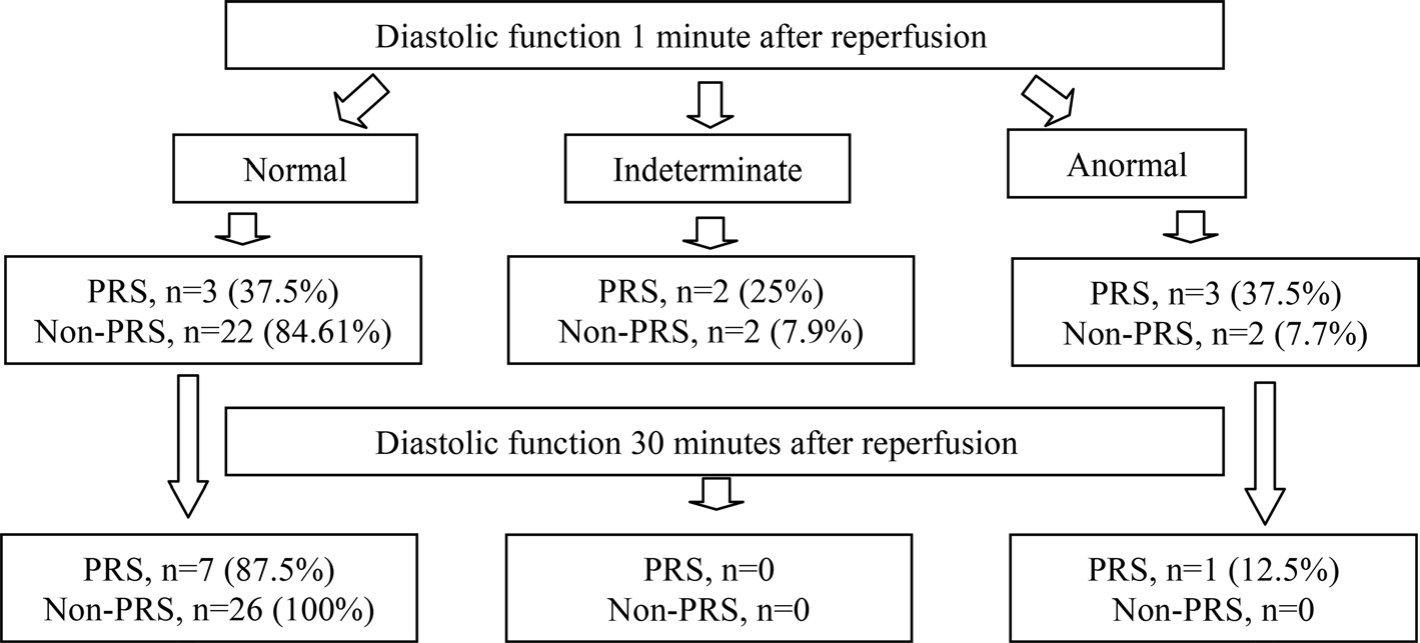
Evaluation of left ventricular diastolic function

After unclamping, the PRS group presented greater systolic/diastolic dysfunction than the non-PRS group (RV dilation ≥ 0.6, TAPSE < 17 mm, LVEF < 53%, and LV diastolic dysfunction (type II). No patient presented diastolic dysfunction type III. Thirty minutes after vascular unclamping, only one patient in the PRS group presented RV dilation and LV diastolic dysfunction. The main findings collected with TEE at 1 and 30 min after graft reperfusion are shown in Fig. 1; Table 2.

**Fig. 2:**
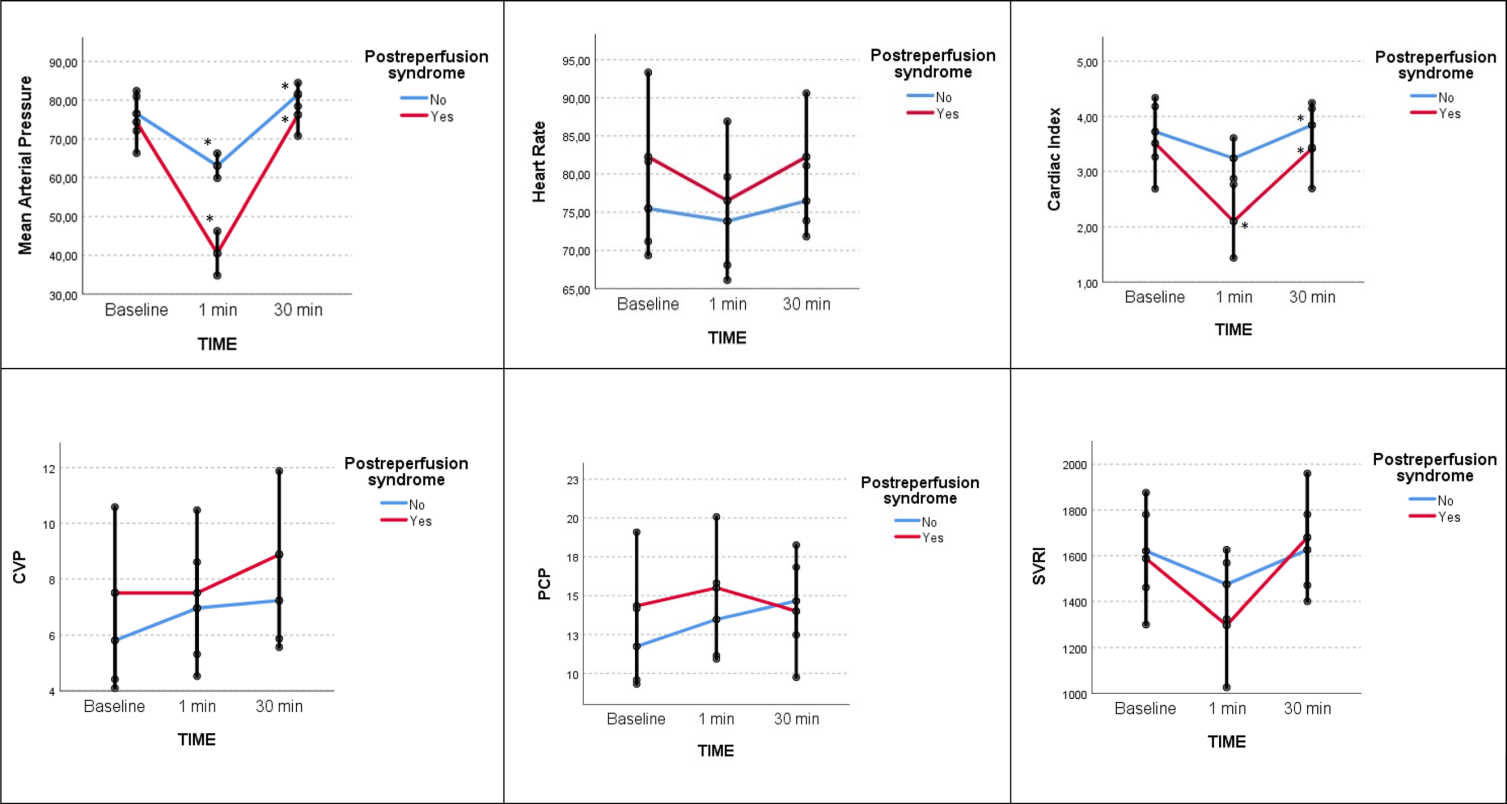
Graphical representation of the evolution of hemodynamic parameters over time and between groups. Marginal estimated means and the error bars that are the confidence intervals 95% (CI 95%) at basal, at 1 and 30 min after graft reperfusion. Statistical differences in the evolutionary pattern of the two groups are marked with an asterisk

Twenty-nine patients were monitored with the Swan Ganz catheter (eight patients in the PRS group and 21 patients in the non-PRS group). MAP decreased significantly in both study groups one minute after vascular unclamping, with a greater reduction in the PRS group. Cardiac output also decreases significantly in the PRS group one minute after vascular unclamping. There were no significant differences between groups in the general evolution of filling pressures (PVC, PCP), HR, and in systemic vascular resistance; although filling pressures increased, and systemic vascular resistance decreased one minute after graft reperfusion. Figure 2; Table 3 show the results.

**Fig. 3:**
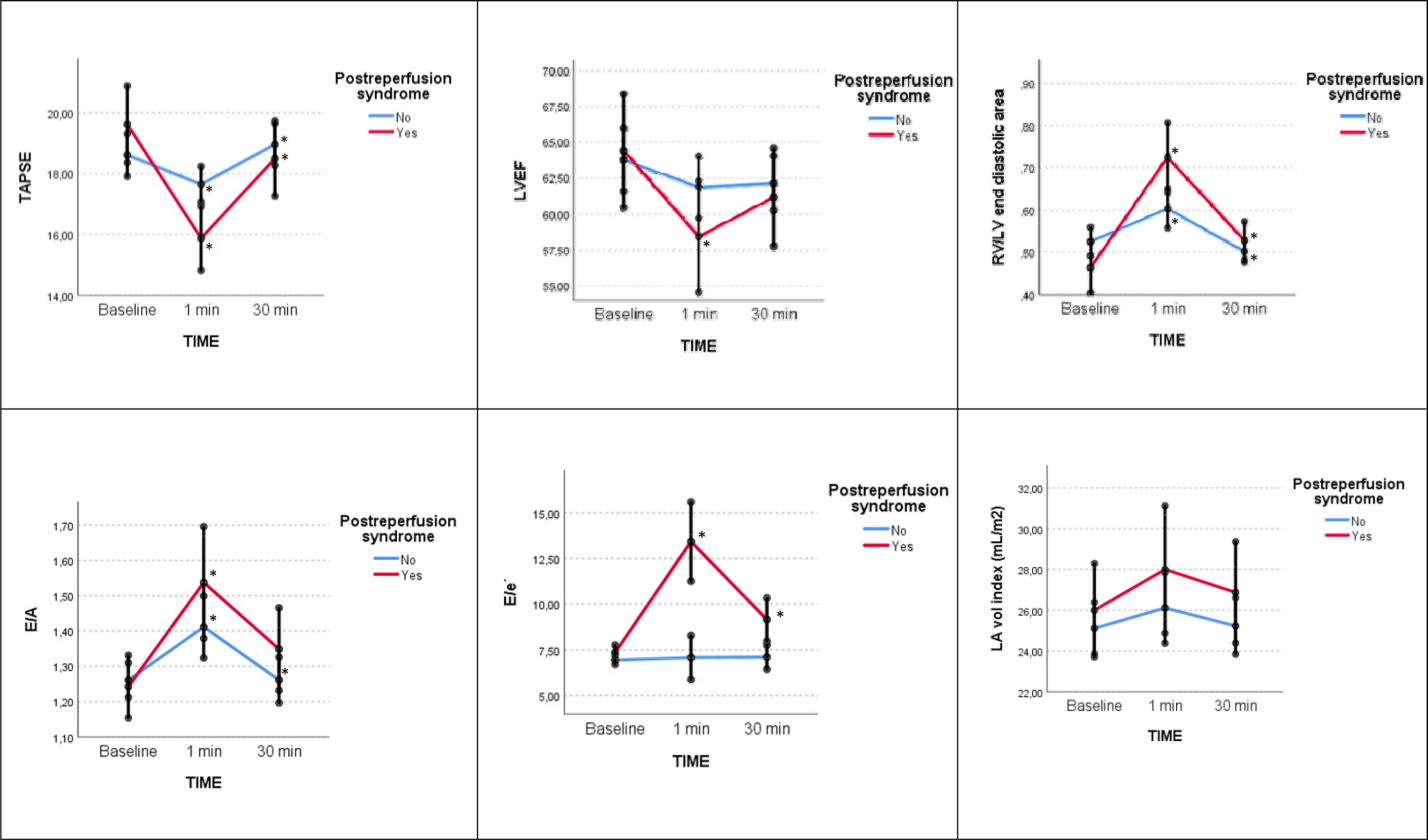
Graphical representation of the evolution of ventricular function over time and between groups. Marginal estimated means and the error bars that are the confidence intervals 95% (CI 95%) at basal, at 1 and 30 min after graft reperfusion. Statistical differences in the evolutionary pattern of the two groups are marked with an asterisk

The evolution of ventricular function over time for two global population showed that the RV was dilated at 1 min after reperfusion (significant increase of 0.170 units (34.5%)), returning to almost baseline values at 30 min (increase with respect to basal value of 0.020 units (4.1%), *p* = 0.000). There were significant differences between groups in the evolutionary pattern at 1 and 30 min after unclamping for RV dilation (*p* = 0.008); thus, the PRS group experienced a more pronounced first increase (56.4%) at 1 min and a subsequent decrease at 30 min (13.8%), leading to a slight, significant increase in RV dilation values between the basal and post-30-min timepoints. The non-PRS group presented an increase of 14.9% at 1 min and of -4.6% at 30 min with respect to basal levels. There were significant differences in the evolutionary pattern over time for TAPSE, LVEF, E/A, E/e´and tricuspid regurgitation (TR) velocity. There were also significant differences between groups in the evolutionary pattern at 1 and 30 min for TAPSE (*p* = 0.014), E/e´ (*p* = 0.000) and TR (*p* = 0.009). The evolution of ventricular dysfunction is shown in Table 4; Fig. 3.

**Table 5:** Dosage of intraoperative vasopressors and postoperative complications in the two groups

		Mean ± SD Number (percentage)	*p-value*
Noradrenaline	Non-PRS	9.56 ± 4.78	0.307
	PRS	7.38 ± 6.38	
Adrenaline	Non-PRS	0.15 ± 0.25	0.39
	PRS	0.08 ± 0.2	
Hospital stay (days)	Non-PRS	15.12 ± 5.53	0.67
	PRS	27 ± 34.18	
Graft rejection	Non-PRS	11.5%	0.94
	PRS	12.5%	
Re-surgery	Non-PRS	7.7%	0.67
	PRS	12.5%	
Re-transplant	Non-PRS	0%	0.06
	PRS	12.5%	
Renal disorder	Non-PRS	26.9%	0.22
	PRS	50%	
Mortality	Non-PRS	3.8%	0.36
	PRS	12.5%	

Hemodynamic instability was observed in one patient after orotracheal intubation due to severe pulmonary hypertension; moderate tricuspid regurgitation allowed to obtain a pulmonary arterial systolic pressure of 58 mmHg (not previously known), and dilation of the RV was observed (RV/LV = 0.9). Given these findings, pulmonary pressure monitoring with a Swan Ganz catheter was employed to confirm the findings. Two patients presented hemodynamic instability in the dissection stage and six patients in the anhepatic stage, which was attributed to hypovolemia (collapse of the ventricular chambers in systole in the TG mid SAX) with systemic vascular resistance between 1100 and 1230 dyn.s/cm^5^). Two patients presented mild right pleural effusion observed by the TEE descending aorta short-axis view, which did not affect the oxygenation of the patients.

Microembolizations were observed in all patients after vascular unclamping in cardiac right cavities.

There were no differences between the groups in the dosage of vasoactive drugs administered during surgery and in postoperative complications (Table 5).

Sixteen patients had esophageal varices, but no complications were associated with the use of the echocardiography probe. No patient experienced (documented) variceal hemorrhage, dental trauma, esophageal or gastric perforation, oropharyngeal trauma, or recurrent laryngeal nerve injury.

## Discussion

The main finding of this study is that cardiac dysfunction was greater in patients with PRS, and that this dysfunction was transient in most patients. The incidence of RV dilation, LV diastolic dysfunction, and biventricular systolic dysfunction was higher in patients with PRS than in those without at one minute of vascular unclamping. By 30 min, all patients had recovered normal LV systolic function, with persistent dilation of the RV and LV diastolic dysfunction in one patient with PRS.

RV dilation occurred in 87.5% of patients with PRS and in 19.2% of the patients without PRS. Some authors [15] have also described RV abnormalities, such as dilation and/or abnormal interventricular septal wall motion (IVSWM) [6], as causes of hypotension after graft reperfusion. During the liver reperfusion stage, many right-sided microemboli and inflammatory mediators flood the systemic circulation from the liver graft, producing a sudden load of cold and acidotic blood. This can lead to right ventricle overload and increase pulmonary circulation (typically, filling pressures increase in this phase). The dilation of the RV also pushes the interventricular septum to the left, inducing transient diastolic dysfunction and decreased preload in the left heart. In fact, in our study, 75% (26.47% of the total) of the patients who presented RV dilation also presented LV diastolic dysfunction one minute after vascular unclamping. This diastolic dysfunction was also observed by Wolf et al. [16] and by Devauchelle et al. [17] when analyzing e’ wave velocity during liver graft reperfusion.

We also observed biventricular systolic dysfunction. 25% of patients with PRS had LV dysfunction and 62.5% had right ventricular systolic dysfunction (TAPSE < 17 mm) one minute after vascular unclamping. We measured TAPSE because it is a very specific parameter of RV systolic function and is less dependent on preload than other markers of ventricular function. However, there are some disadvantages; the measurement is angle dependent, and the displacement in the ME 4 C view is representative of total RV function only if there are no regional RV wall motion abnormalities [18].

Decreased left heart preload can lead to a decrease in systemic systolic blood pressure. This can have two components: the first cardiac, as described above, and a second component due to the liberation of the solute from the liver into the systemic circulation, sustaining the hypotension. To decrease the air embolism, hypothermia, and ischemic metabolites from the donor liver, an albumin hot solution was injected into the donor liver by our surgery team, discarding the effluent immediately before graft reperfusion. Even so, we observed right sided microemboli in all patients, although we were unable to document the impact of less or more air embolization on the degree of cardiac dysfunction. We also normalize calcium chloride, potassium, bicarbonate, and glucose levels before reperfusion of the donated liver.

There are no conclusive results on the prognostic impact of cardiac dysfunction. Many of our patients recovered normal cardiac function by 30 min after unclamping, and we did not observe differences in postoperative complications between groups. Similarly, other authors did not obtain any prolonged cardiac dysfunction as a common problem during liver transplantation and concluded that an understanding of the physiological changes that occur during transplantation should allow the anesthesiologist to correct many factors that might cause hemodynamic instability [16]. Marella et al. obtained similar results and concluded that LV diastolic dysfunction did not affect post-transplant outcomes [19]. Nevertheless, other reports have shown that LV diastolic dysfunction is associated with an increased risk of graft rejection, graft failure, and mortality [20–23].

This present study has some limitations. The main limitation was the small sample size; nevertheless, we could still find some differences in our population study. Prospective studies should be performed to confirm the cardiac dysfunction after liver graft reperfusion, and its repercussion on postoperative complications. On the other hand, we could be underestimating the degree of the LV diastolic dysfunction because TEE underestimates both the diameter and volume of the LA compared to TTE. In any case, we performed a Simpson biplane estimation of the LA volume in TEE, which is the best measure to estimate the LA volume from TTE with a mean difference of -6 ml [9]. Finally, we did not determine the diastolic function of the RV in our patients and only measured the ratio RV/LV end diastolic area and TAPSE. Given the anatomical disposition of RV, the alignment of the Doppler cursor with the RV free wall is usually not permitted on ME views in TEE, as the cursor and tissue motion are in distinctly different orientations. Consequently, RV diastolic dysfunction is less frequently documented.

We can affirm that TEE monitoring offers some advantages at the reperfusion stage as cardiac function can be monitored in real time, allowing an optimal outcome after vascular unclamping. Although we did not observe complications associated with the TEE probe, it is necessary to consider that serious complications such as esophageal perforation have been reported in liver transplant recipients [24]. Therefore, careful insertion and mobilization of the probe during exploration should be a priority.

## Conclusion

PRS in OLT is related to RV dysfunction with a certain degree of LV dysfunction, although this cardiac dysfunction recovers in most patients at 30 min post vascular unclamping, without postoperative clinical repercussions.
